# Manual Hippocampal Subfield Segmentation Using High-Field MRI: Impact of Different Subfields in Hippocampal Volume Loss of Temporal Lobe Epilepsy Patients

**DOI:** 10.3389/fneur.2018.00927

**Published:** 2018-11-20

**Authors:** Jose Eduardo Peixoto-Santos, Luciana Estefani Drumond de Carvalho, Ludmyla Kandratavicius, Paula Rejane Beserra Diniz, Renata Caldo Scandiuzzi, Roland Coras, Ingmar Blümcke, Joao Alberto Assirati, Carlos Gilberto Carlotti, Caio Cesar Marconato Simoes Matias, Carlos Ernesto Garrido Salmon, Antonio Carlos dos Santos, Tonicarlo R. Velasco, Marcio Flavio D. Moraes, Joao Pereira Leite

**Affiliations:** ^1^Department of Neurosciences and Behavioral Sciences, Ribeirao Preto Medical School, University of Sao Paulo, Ribeirao Preto, Brazil; ^2^Neuropathology Institute, University Hospitals Erlangen and Friedrich-Alexander University of Erlangen-Nuremberg, Erlangen, Germany; ^3^Department of Physiology and Biophysics, Federal University of São João del Rey, Divinópolis, Brazil; ^4^Department of Clinical Medicine, Federal University of Pernambuco, Recife, Brazil; ^5^Department of Surgery and Anatomy, Ribeirao Preto Medical School, University of Sao Paulo, Ribeirao Preto, Brazil; ^6^Department of Physics and Mathematics, Faculty of Philosophy, Science and Languages of Ribeirao Preto, University of Sao Paulo, Ribeirao Preto, Brazil; ^7^Department of Internal Medicine, Ribeirao Preto Medical School, University of Sao Paulo, Ribeirao Preto, Brazil; ^8^Department of Physiology and Biophysics, Center for Technology and Research in Magneto-Resonance, Federal University of Minas Gerais, Belo Horizonte, Brazil

**Keywords:** high field MRI, hippocampal subfield volumetry, neuronal density, hippocampal sclerosis, 4.7T, *ex vivo* imaging

## Abstract

In patients with temporal lobe epilepsy (TLE), presurgical magnetic resonance imaging (MRI) often reveals hippocampal atrophy, while neuropathological assessment indicates the different types of hippocampal sclerosis (HS). Different HS types are not discriminated in MRI so far. We aimed to define the volume of each hippocampal subfield on MRI manually and to compare automatic and manual segmentations for the discrimination of HS types. The T2-weighted images from 14 formalin-fixed age-matched control hippocampi were obtained with 4.7T MRI to evaluate the volume of each subfield at the anatomical level of the hippocampal head, body, and tail. Formalin-fixed coronal sections at the level of the body of 14 control cases, as well as tissue samples from 24 TLE patients, were imaged with a similar high-resolution sequence at 3T. Presurgical three-dimensional (3D) T1-weighted images from TLE went through a FreeSurfer 6.0 hippocampal subfield automatic assessment. The manual delineation with the 4.7T MRI was identified using Luxol Fast Blue stained 10-μm-thin microscopy slides, collected at every millimeter. An additional section at the level of the body from controls and TLE cases was submitted to NeuN immunohistochemistry for neuronal density estimation. All TLE cases were classified according to the International League Against Epilepsy's (ILAE's) HS classification. Manual volumetry in controls revealed that the dentate gyrus (DG)+CA4 region, CA1, and subiculum accounted for almost 90% of the hippocampal volume. The manual 3T volumetry showed that all TLE patients with type 1 HS (TLE-HS1) had lower volumes for DG+CA4, CA2, and CA1, whereas those TLE patients with HS type 2 (TLE-HS2) had lower volumes only in CA1 (*p* ≤ 0.038). Neuronal cell densities always decreased in CA4, CA3, CA2, and CA1 of TLE-HS1 but only in CA1 of TLE-HS2 (*p* ≤ 0.003). In addition, TLE-HS2 had a higher volume (*p* = 0.016) and higher neuronal density (*p* < 0.001) than the TLE-HS1 in DG + CA4. Automatic segmentation failed to match the manual or histological findings and was unable to differentiate TLE-HS1 from TLE-HS2. Total hippocampal volume correlated with DG+CA4 and CA1 volumes and neuronal density. For the first time, we also identified subfield-specific pathology patterns in the manual evaluation of volumetric MRI scans, showing the importance of manual segmentation to assess subfield-specific pathology patterns.

## Introduction

Epilepsy affects 0.6–1.5% of the world's population (1). Temporal lobe epilepsy (TLE) is the most frequent focal epilepsy in adults, and drug-resistance is common in these patients (2). Resection of the epileptogenic zone, when indicated by the presurgical evaluation, is the main treatment for drug-resistant cases (3). Magnetic resonance imaging (MRI) is fundamental to the presurgical definition of the epileptogenic focus (4–6). In most TLE cases, MRI often reveals hippocampal atrophy in T1-weighted imaging and increased signal in T2-weighted imaging (5, 7–10).

The postsurgical histological assessment of the resected hippocampus frequently reveals hippocampal sclerosis (HS), characterized by differential neuronal cell loss and gliosis (11–17). In most cases, neuron loss is severe in CA1 and CA4 and moderate in CA3, while the subiculum and CA2 are preserved (14). However, some patients present neuronal loss circumscribed to only CA1 or CA4 (14). Although quantitative studies correlated the severity of neuronal loss and gliosis to the degree of hippocampal atrophy measured in MRI (7, 9, 18, 19), there is no consent about which hippocampal subfield has a more significant impact on the hippocampal atrophy. Moreover, it is essential to evaluate the contribution of each hippocampal subfield for the hippocampal volume in control cases, to better define volume loss in TLE cases.

Since different HS patterns are related to different postsurgical prognostic predictions (14, 20–22), the possibility to detect these patterns in the presurgical MRI can guide the surgical decision better. However, the accurate labeling of each hippocampal subfield, corrected by histology, is a crucial first step for the development of automatic *in vivo* subfield evaluation. We aimed to evaluate the volume of each hippocampal subfield by *ex vivo* MRI of the hippocampal formation of autopsy cases without neurological diseases, compare volumetric differences between controls and hippocampi resected from drug-resistant TLE cases with different HS types, and to compare manual and automatic segmentation identification of these HS type patterns.

## Materials and methods

### Patients and clinical data

Patients with drug-resistant epilepsy (TLE, *n* = 24) were evaluated at the Epilepsy Surgical Centre of Ribeirao Preto Medical School, University of Sao Paulo (Brazil), according to standard protocols (23, 24). Inclusion criteria were a diagnosis of TLE not involving tumor, dysplasia, or other cortical malformations and age between 18 and 70 years.

Control hippocampi (*n* = 14) were obtained from age-matched autopsy cases without a history of neurological diseases or sign of brain pathologies in postmortem pathological evaluation. All control cases were obtained within < 12 h *postmortem*.

Medical records of all cases were assessed for clinical data analysis. The clinical variables investigated were the age at death and cause of death for control and age at surgery, epilepsy duration, age at epilepsy onset, seizure frequency for TLE patients, and variables related to epilepsy. This study was registered in the Brazilian Health Ministry and was approved by our local ethics committee (process HCRP 7200/2016).

### Tissue collection

For the control cases, hippocampi from both sides were removed during the autopsy procedure. The brain was held with its base facing up, and an incision was made, in the parahippocampal gyrus (perpendicular to the gyrus' long axis, ~3 cm posterior to the mesencephalon), until the lateral ventricle was evident. The ventricle was dissected by cutting along the occipitotemporal sulcus (i.e., between the medial occipitotemporal gyrus and the lateral occipitotemporal gyrus), from the posterior temporal lobe to the temporal pole, exposing the hippocampus. After the base of the ventricle had been removed, the hippocampi were fixed and used for imaging. En bloc resection was performed in the TLE cases for the treatment of drug-resistant epilepsy, and sections from the hippocampal body were fixed in 4% buffered formaldehyde.

After 8 days of fixation, the entire hippocampus of the control cases went through imaging procedure in a 4.7T MR scanner. After scanning at 4.7T, the whole hippocampus was sectioned, and sections from the hippocampal body from control cases, as well as from TLE cases, went through imaging in a 3T scanner.

### MRI acquisition

#### *In vivo* (clinical resolution) imaging for automatic segmentation

Presurgical three-dimensional (3D) T1-weighted sequences from the TLE cases were retrieved from the medical archives. The volumetric T1 images were captured in a 3.0T Philips Achieva X-series MR with an eight elements phase-array head coil. The 3D single-shot T1-weighted images were captured, with TE = 3.2 ms, TR = 7 ms, 8° flip angle, inversion pulse = 900 ms; shot interval = 2,500 ms; voxel size = 1 mm^3^; FOV = 240 × 240 mm. The imaging time was 4.5 min.

#### *Ex vivo* (high resolution) imaging for manual segmentation

Formalin-fixed hippocampi from control cases were submitted for MRI in a 4.7T NMR system (OxfordSystems) controlled by a UNITY Inova-200 imaging console (Varian). The imaging protocol consisted of coronal T2-weighted spin echo multi-slice images (TE = 50 ms; TR = 3,000 ms; resolution of 0.01 × 0.02 mm; 0.1 mm slice thickness; 45 contiguous coronal slices; FOV = 240 × 180 mm) that were obtained for the structural analysis. The acquisition time was 1 h and 30 min.

For the imaging of the hippocampal body, fixed sections from TLE and control cases were submitted for MRI in the same 3.0T Philips Achieva X-series machine as the presurgical MRI, with a finger quadrature coil. The sequence used for structural assessment was the 50 ms echo from a turbo spin-echo relaxometry sequence (TE = 25–125 ms; TR = 3,400 ms; resolution of 0.4 × 0.4 mm; 0.5 mm slice thickness; 54 coronal slices; FOV = 25 × 25 mm; 30 repetitions). The imaging time was 2 h and 18 min.

### Histological evaluation

After the MRI procedure, the hippocampi were sectioned and embedded in paraffin. For the whole hippocampus evaluation, all sections were cut, and 10-μm coronal sections were collected at every 100 μm of the hippocampal length. For the evaluation of the neuronal density, a section at the level of the hippocampal body was submitted for NeuN immunohistochemistry.

To better define the hippocampal subfields on MRI, one section at each millimeter was stained with Luxol Fast Blue. The sections were rehydrated to 95% alcohol, incubated in Solvent Blue 38 (Sigma, Germany) solution overnight at 60°C, washed and immersed in 0.05% lithium carbonate solution, washed, counterstained with Cresyl Violet (Sigma, Germany) solution, dehydrated, and mounted with Krystalon.

For neuronal density, a published protocol for NeuN immunohistochemistry was used (13, 25). From all cases, four control cases had no immunopositivity for NeuN; thus only 8 of the 14 controls were evaluated for neuronal density. We collected representative images from each subfield with Zeiss AxioImager M1. Neuronal density was estimated using Abercrombie's technique (26), as follows: neurons were counted in five square regions of interest (ROIs) of 10,000 μm^2^ for each subfield, and neuronal density was obtained with the formula

$$\begin{array}{l} Density=\;\frac{neuron\;count*\left(\frac{section\;thickness}{section\;thickness+cell\;diameter}\right)}{total\;area*section\;thickness} \end{array}$$

Cell density was then converted to z-score for statistical analysis.

Based on the International League Against Epilepsy's (ILAE's) task force classification (14), TLE patients were subdivided into those with HS type 1 (TLE-HS1) and those with HS type 2 (TLE-HS2).

### Hippocampal volumetry

#### Freesurfer hippocampal subfield automatic detection

The presurgical volumetric images were converted from DICOM (^*^.dcm) to MNI (^*^.mnc), and FreeSurfer Version 6.0 was used to automatically detect the grey matter and white matter volumes of all brain structures, as described elsewhere (27, 28). After the delineation of all brain structures, the subroutine of hippocampal subfield detection (29) was performed with the following command line:

recon-all -s <file_name> -hippocampal-subfields-T1

With this command, two separate TXT files with left and right hippocampal subfield volumes, as well as whole hippocampal volume, were available for statistical analysis. From the subfield routine, we selected the volumes of subiculum, CA1, CA3+CA2 (delineated together by the algorithm), and whole hippocampus. To better match our DG+CA4 category (see Section Hippocampal Subfield Definition and Supplementary Figure 1), we added the values of the segmented regions CA4 and GC-ML-DG. For FreeSurfer automatic evaluation, the controls used were the contralateral hippocampi of TLE cases without evidence of HS (23 of the 24 cases). These cases had volumetry similar to normal values from a previous study (9) and had similar volume to age- and sex-matched healthy voluntaries (*n* = 4, not shown in the study) in all subfields (*p* > 0.05).

#### Manual hippocampal subfield delineation

For the 4.7T experiment, the volumetry of the hippocampal subfields was done manually with the MeVisLab software (MeVis Medical Solution AG). The images were opened in the software and evaluated with a homemade freehand routine, which was developed at CTPMAG (Federal University of Minas Gerais, Brazil). Briefly, the ^*^.fdf images generated by the scanner were converted to ^*^.img files with a MATLAB converter, and these files were then opened in MeVisLab with the ImageLoad module. Once loaded, the OrthoView2D module was used to delineate the subfields in freehand mode with the CSOFreehandProcessor. The CSOManager module was used to remove any wrong labels. After the delimitation, the masks with the volumes of every subfield were saved with the ImageSave module in the RAW format for posterior quantification of the volume in the MATLAB R2014b software (MathWorks). For this step, the original ^*^.img MRI file was loaded together with the individual masks of each subfield (in ^*^.raw format), and the volume (in cm^3^) was measured. The borders between subfields throughout the hippocampus are shown in Supplementary Figure 1.

For the 3T experiment, the volumetry of the hippocampal subfields at the level of the hippocampal body was done manually with MINC Tools (BIC, McGill, Canada). Briefly, the T2-weighted ^*^.dcm images were converted to ^*^.mnc with dcm2minc command in Terminal (^*^.nii and ^*^.mgh are also accepted formats) and opened with the Display command, also in Terminal. In the Segmenting menu, each subfield was manually delineated with freehand brush in a specific color label (Supplementary Figure 2). Labels were assigned with Set Paint Label command followed by the label numeric code, and the brush size was set at 0.1 with XY Radius command. The subfield boundaries were delineated with the chosen label, and the contour was filled by placing the cursor inside the delineated area followed by the command Label Fill. After finishing each subfield, the volume was directly measured with the Calculate Volume command. After all subfields were marked and measured, all labels were saved in a single file with the Save Labels.mnc command in the File menu. More details can be found at http://www.bic.mni.mcgill.ca/software/Display/Display.html.

### Hippocampal subfield definition

The delineation of hippocampal subfields throughout the hippocampal regions (head, body, and tail) followed Duvernoy's sectional anatomy definitions (30), and the subdivisions were as follows: dentate gyrus (DG, comprising molecular layers, granule cell layer, and the polymorphic layer) and CA4 grouped (DG+CA4); CA3; CA2; CA1; and subiculum. The grouping of DG and CA4 was done, since segmenting and separating these subfields in MRI with confidence was not feasible. Neuronal density was analyzed in CA4, CA3, CA2, CA1, and in the subiculum.

### Statistics

Differences in clinical data were evaluated with Exact's test (categorical) and Student's *t*-test or Mann–Whitney's test (continuous). Two-Way ANOVA with sex as a co-factor were performed to compare hippocampal subfield volumes and neuron density of controls, TLE-HS1, and TLE-HS2. Spearman's correlation test was used to evaluate the associations between hippocampal volume and neuronal density. Intra-rater correlation coefficients were calculated between the initial manual volumetric assessment (JP-S) and the final volumetry (JP-S with anatomical corrections from RC). Cohen's kappa coefficient was estimated for HS classification (JP-S and JPL). For statistical comparisons, all volumetric and neuronal density data was z-scored. We performed the statistical analysis with the SigmaPlot 11 software, graphs in R, and results were considered significant at *p* < 0.05.

## Results

### Clinical and histological data

Following the ILAE classification, 92% (22) of the TLE patients had HS type 1 (TLE-HS1) while two patients had HS type 2 (8%). No control case presented with HS. The inter-rater Cohen's kappa coefficient for HS classification indicated substantial agreement between evaluators (κ = 0.84). All groups were age-matched (control = 48 ± 15, TLE-HS1 = 41 ± 11, TLE-HS2 = 40 ± 14). The control group was different from the TLE groups with regard to sex (control = 92% male, TLE-HS1 = 41% male, TLE-HS2 = 50%; exact test, *p* = 0.008). The TLE-HS1 and TLE-HS2 groups had no differences with regard to initial precipitating injury (IPI) occurrence, IPI type, age at IPI, age at seizure recurrence, seizure frequency, seizure generalization, seizure clustering, the occurrence of status epilepticus, familiar history of epilepsy, surgical outcome, or presence and type of other presurgical MRI findings (Table 1). In the control group, 67% died from heart-related diseases and the remaining by septic shock. The time between death and fixation of the samples was on average 3.96 h, ranging from 3.1 to 12 h after death. Logistic regression confirmed the lack of differences between TLE-HS1 and TLE-HS2 with regard to the clinical variables (*p* > 0.096).

**Table 1:** Clinical data from the control and TLE patients^*^.

		**Control**	**TLE-HS1**	**TLE-HS2**	***p***
Age^**^ (years)		48 ± 15^***^	41 ± 11	40 ± 14	0.179^a^
Sex (male)		13 (92%)	9 (41%)	1 (50%)	0.008^b^
IPI age (years)		–	10 ± 9	3 ± 3	0.320^c^
IPI	No IPI	–	9 (41%)	1 (50%)	0.943^b^
	Febrile seizure	–	7 (32%)	1 (50%)	
	Afebrile seizure	–	2 (9%)	0 (0%)	
	TBI	–	2 (9%)	0 (0%)	
	Meningitis	–	2 (9%)	0 (0%)	
Seizure recurrence (years)		–	18 ± 12	10 ± 6	0.343^d^
Seizure frequency (monthly)		–	7 ± 10	17 ± 18	0.178^c^
Seizure generalization		–	10 (45%)	1 (50%)	0.902^b^
Seizures in clusters		–	13 (59%)	1 (50%)	0.803^b^
Status epilepticus (occurrence)		–	6 (27%)	1 (50%)	0.498^b^
Familiar history of epilepsy (positive)		–	13 (59%)	2 (100%)	0.253^b^
Surgical Outcome	ILAE1	–	14 (64%)	1 (50%)	0.742^b^
	ILAE2	–	2 (9%)	0 (0%)	
	ILAE3	–	2 (9%)	0 (0%)	
	ILAE4	–	3 (14%)	1 (50%)	
	ILAE5	–	1 (4%)	0 (0%)	
Other MRI findings	No other pathology	–	10 (45%)	1 (50%)	0.732^b^
	Cerebral/cerebellar atrophy		6 (27%)	0 (0%)	
	Extratemporal calcification		2 (9%)	1 (50%)	
	Diffuse microangiopathy		1 (4%)	0 (0%)	
	Temporal pole blurring		2 (9%)	0 (0%)	
	Gliotic lacunae in the Caudate		1 (4%)	0 (0%)	
	Hypophysis hypertrophy		1 (4%)	0 (0%)	

* *Only cases with high-resolution MRI are shown in this clinical table*.

** *Age at surgery for TLE and age of death for controls*.

*** Average ± standard deviation.

a *One-way ANOVA*.

b *Exact test*.

c *Mann–Whitney test*.

d *Student's t-test*.

**Figure 1:**
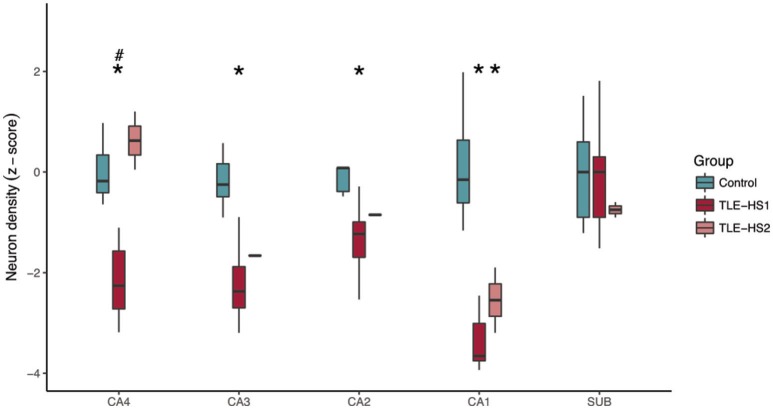
Neuronal density, shown as z-score, in the hippocampal subfields of control cases (green boxplots) and TLE cases with type 1 HS (TLE-HS1, dark red boxplots) or type 2 HS (TLE-HS2, light red boxplots). TLE-HS1 patients had lower neuronal density than controls in all measured hippocampal subfields but the subiculum, whereas TLE-HS2 had only lower neuronal density in CA1. TLE-HS1 also had lower neuronal density than TLE-HS2 in DG+CA4. The asterisks indicate difference from control cases. DG, dentate gyrus; SUB, subiculum; HIP, all hippocampal subfields.

Owing to the sex misbalance between groups, a two-way ANOVA factoring group and sex was used to evaluate differences between neuronal density and volumetric measurements. The TLE-HS1 cases had lower neuronal density than controls in CA4, CA3, CA2, and CA1 (*p* < 0.001; Figure 1). The TLE-HS1 cases had a lower neuronal density than TLE-HS2 patients in CA4 (*p* < 0.001). Patients with TLE-HS2 had lower neuronal density than controls only in CA1 (*p* = 0.003). All groups had the same neuronal density in the subiculum (*p* = 0.371). Sex had no significant effect on neuronal density (*p* ≥ 0.163).

### Freesurfer hippocampal volumetry at 3T

**Figure 2:**
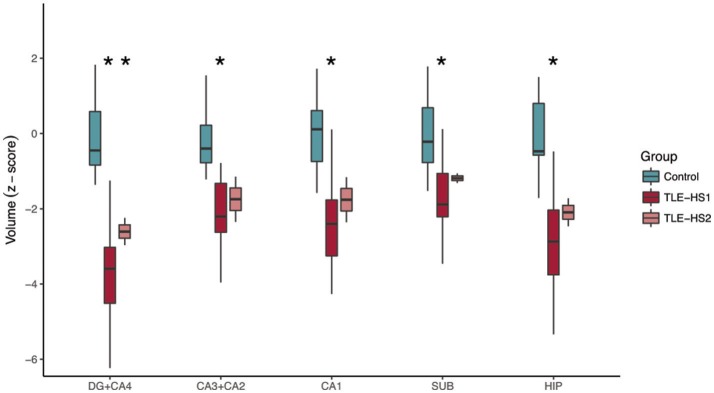
Automatic subfield evaluation with FreeSurfer of control cases (i.e., the contralateral hippocampi without evidence of HS; green boxplots) and TLE cases with type 1 HS (TLE-HS1, dark red boxplots) or type 2 HS (TLE-HS2, light red boxplots). TLE-HS1 patients had lower volumes, compared with controls, in all subfields, whereas TLE-HS2 had lower volume only in DG+CA4. The asterisks indicate difference from control cases. DG, dentate gyrus; SUB, subiculum; HIP, all hippocampal subfields.

The automatic evaluation of hippocampal subfields showed widespread volume loss in all hippocampal subfields of TLE-HS1 cases, when compared with controls (Figure 2, *p* < 0.001). Patients with TLE-HS2 had only lower volume, when compared with controls, in DG+CA4 (*p* = 0.032). Automatic subfield detection was unable to differentiate TLE-HS1 from TLE-HS2 (*p* ≥ 0.667). To test whether the lack of difference between HS 1 and HS 2 was due to the low number of HS 2 cases, we evaluated the presurgical MRI from four additional HS 2 cases (from which we did not have the high-resolution 3T *ex vivo* MRI). Similar to the previous results, TLE-HS2 remained indiscernible from TLE-HS1 (*p* ≥ 0.729; Supplementary Figure 3), and both had lower volumes in all subfields when compared with controls (*p* ≤ 0.031).

### Manual hippocampal volumetry at 3T and 4.7T

**Figure 3:**
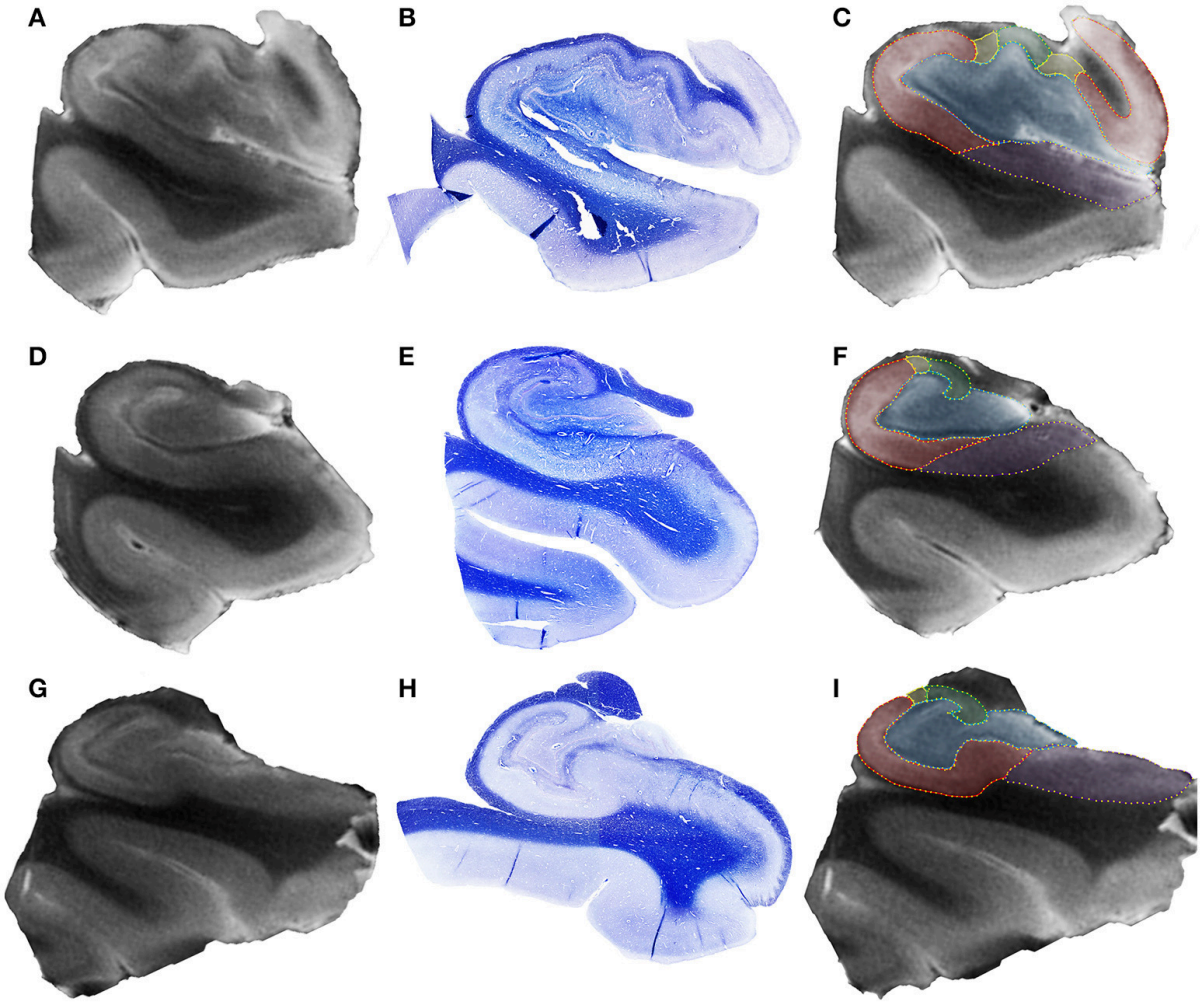
Representative 4.7T coronal images from hippocampal head **(A)**, hippocampal body **(D)**, and hippocampal tail **(G)** of a control case. In the middle row **(B,E,H)**, see the respective histological section stained with Luxol Fast Blue. The right column **(C,F,I)** shows the delineation of hippocampal subfields in each hippocampal region, following the code: blue, dentate gyrus + CA4; green, CA3; yellow, CA2; red, CA1; purple, subiculum.

**Figure 4:**
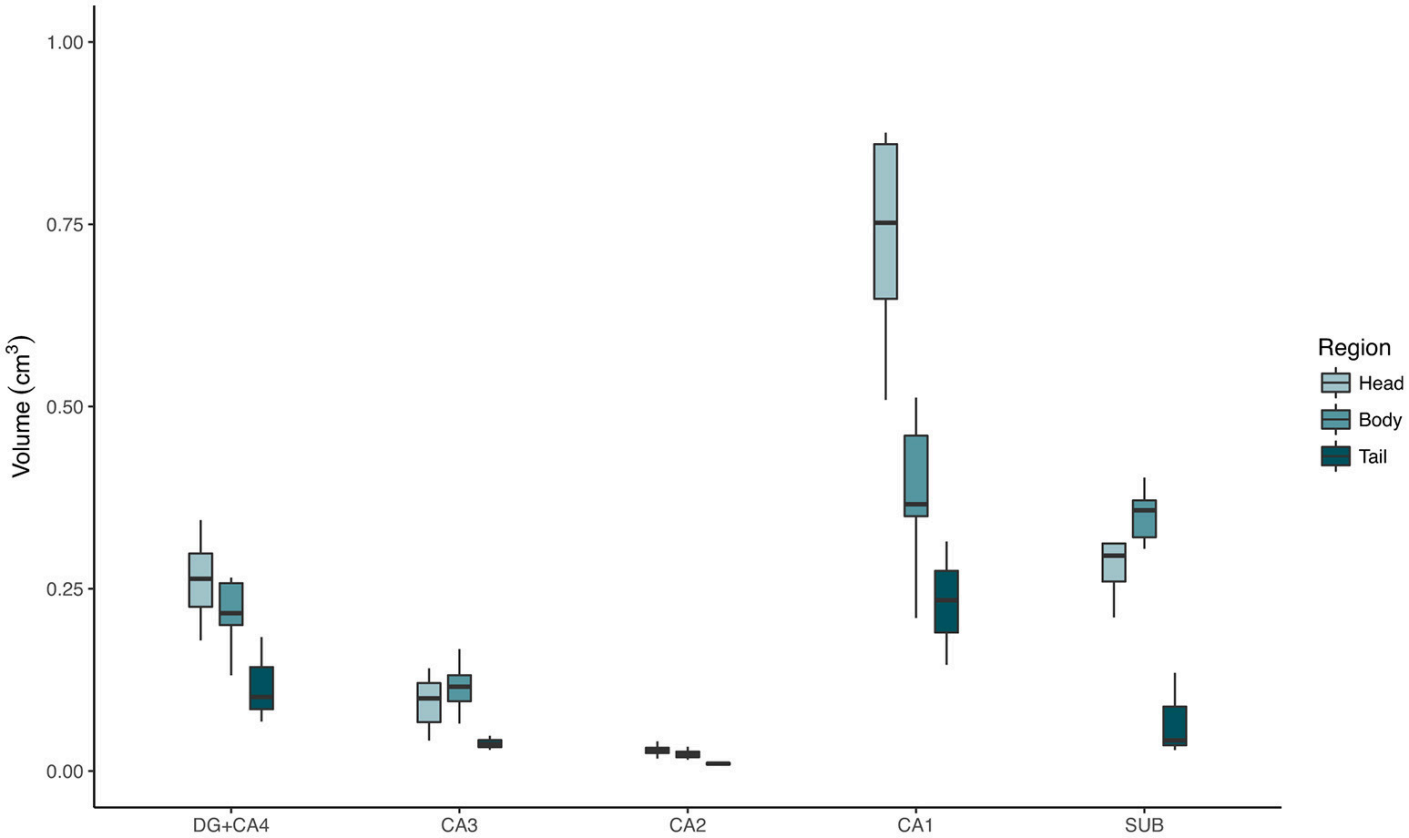
Absolute volumes of each hippocampal subfield, from control cases, in the hippocampal head (light green boxplots), body (medium green boxplots), and tail (dark green boxplots), measured at 4.7T MRI. DG, dentate gyrus; SUB, subiculum.

The evaluation of control hippocampi at 4.7T indicated that DG+CA4, CA1, and the subiculum together correspond to 89.67% of all hippocampal volume (Table 2 and Figures 3, 4). Moreover, the subfields present differential contributions to the hippocampal volume, depending on the level of the hippocampus. In the head of the hippocampus, CA1 makes up to 49.90% of the total head volume, whereas in the body, CA1 accounts for only 35.26% of the hippocampal body volume. The intra-rater correlation coefficients (ICCs) for each subfield are presented in Supplementary Table 1.

**Table 2:** Hippocampal subfield volumes in the head, body, tail, and whole hippocampus.

**Region**		**Volume (cm^3^)**	**Volume (%)**	
			Relative to total volume^*^	Relative to regional volume^**^
DG+CA4	Head	0.26 ± 0.05	8.71 ± 1.76	18.46 ± 3.73
	Body	0.24 ± 0.09	7.97 ± 3.07	21.49 ± 8.28
	Tail	0.12 ± 0.06	3.90 ± 1.79	24.82 ± 12.55
	Total	0.62 ± 0.07	20.58 ± 2.79	
CA3	Head	0.09 ± 0.03	3.14 ± 1.15	6.64 ± 2.44
	Body	0.12 ± 0.03	3.84 ± 1.06	10.35 ± 2.85
	Tail	0.04 ± 0.01	1.26 ± 0.32	8.00 ± 2.04
	Total	0.25 ± 0.03	8.23 ± 1.31	
CA2	Head	0.03 ± 0.01	0.95 ± 0.26	2.01 ± 0.55
	Body	0.02 ± 0.01	0.82 ± 0.27	2.20 ± 0.72
	Tail	0.01 ± 0.002	0.33 ± 0.08	2.10 ± 0.51
	Total	0.06 ± 0.01	2.10 ± 0.32	
CA1	Head	0.71 ± 0.12	23.55 ± 3.93	49.90 ± 8.33
	Body	0.39 ± 0.08	13.08 ± 2.73	35.26 ± 7.35
	Tail	0.24 ± 0.10	7.96 ± 3.23	50.64 ± 20.56
	Total	1.35 ± 0.10	44.59 ± 7.10	
SUB	Head	0.33 ± 0.11	10.85 ± 3.77	22.99 ± 7.99
	Body	0.34 ± 0.12	11.38 ± 3.99	30.70 ± 10.75
	Tail	0.07 ± 0.06	2.27 ± 1.91	14.43 ± 12.15
	Total	0.74 ± 0.10	24.50 ± 4.73	

* *Percentage relative to total hippocampal volume*.

** *Percentage of the subfield in the hippocampal subdivisions (i.e., head, body, and tail)*.

*DG, dentate gyrus; SUB, subiculum*.

**Figure 5:**
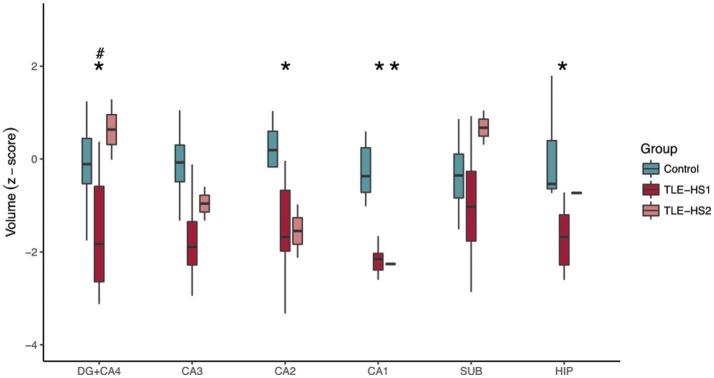
Z-scored hippocampal subfield volumes (at body level) of control cases (green boxplots) and TLE patients with type 1 HS (TLE-HS1, dark red boxplots) or HS type 2 (TLE-HS2, light red), measured in 3T MRI. TLE-HS1 has lower volumes than controls in DG+CA4, CA2, CA1, and in the whole hippocampal volume, whereas TLE-HS2 has only lower volume in CA1. TLE-HS1 also has lower volume than TLE-HS2 in the DG+CA4. The asterisks indicate difference from controls, and the hash/pound sign indicates difference from TLE-HS1. DG, dentate gyrus; SUB, subiculum; HIP, all hippocampal subfields.

The evaluation of hippocampal volume at 3 T showed reduced volumes of DG+CA4 (*p* = 0.012), CA2 (*p* = 0.038), CA1 (*p* < 0.001), and the whole hippocampal volume (*p* < 0.001; Figure 5) of TLE-HS1 patients, when compared with control cases. The TLE-HS2 cases had only low CA1 volume, when compared with controls (*p* = 0.002). In DG+CA4, TLE-HS2 had higher volume than TLE-HS1 (*p* = 0.016). Sex had no effect on subfield volume (*p* ≥ 0.313).

### Correlations between histology and hippocampal volume

**Figure 6:**
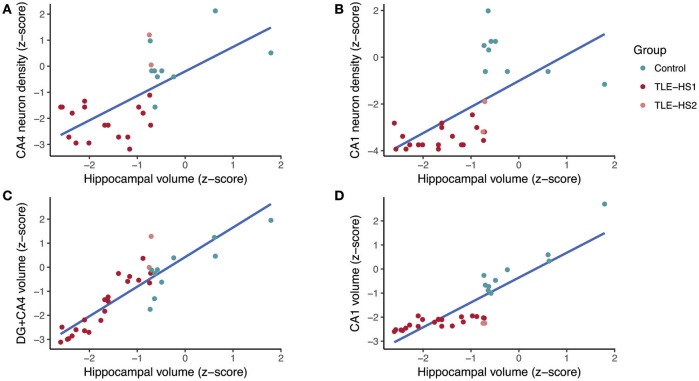
Regression fit between whole hippocampal volume and neuronal density in CA4 **(A)**, neuronal density in CA1 **(B)**, volume of the dentate gyrus + CA4 **(C)**, and volume of CA1 **(D)** in controls (green dots) and TLE patients (red dots). DG, dentate gyrus.

The subfield volume and neuronal density correlated positively in CA1 (*r* = 0.658, *p* < 0.001) and CA3 (*r* = 0.593, *p* < 0.001), and the volume of DG+CA4 correlated positively with CA4 neuronal density (*r* = 0.461, *p* = 0.010). The whole hippocampal volume correlated positively with neuronal density in CA4 (*r* = 0.629, *p* < 0.001; Figure 6A), CA3 (*r* = 0.826, *p* < 0.001), and CA1 (*r* = 0.777, *p* < 0.001; Figure 6B). The hippocampal volume also correlated with DG+CA4 volume (*r* = 0.876, *p* < 0.001; Figure 6C), CA3 (*r* = 0.808, *p* < 0.001), and CA1 volume (*r* = 0.857, *p* < 0.001; Figure 6D). When analyzing only the TLE cases, the neuronal density in CA1 correlated positively with the hippocampal volume (*r* = 0.481, *p* = 0.027). The whole hippocampal volume of TLE also correlated positively with the volumes of DG+CA4 (*r* = 0.933, *p* < 0.001), CA3 (*r* = 0.731, *p* < 0.001), and CA1 (*r* = 0.630, *p* < 0.001).

## Discussion

The hippocampal formation, with its various subfields and connectivity, is a major hub for higher cognitive function, such as memory consolidation and learning, spatial navigation, and emotional expression/affective behavior (31, 32). The role of the hippocampus in long-term, declarative memory formation was first established with studies in a TLE patient who underwent bilateral mesial temporal lobectomy and suffered, as a consequence, anterograde amnesia for explicit contents (33, 34). Since then, several studies have shown that each hippocampal subfield has a differential impact on memory formation (12, 14, 21, 35–37). For instance, animal models have shown that CA1 is strongly related to spatial memory (38), CA2 to social memory (39), and CA3 with learning processes (40). Moreover, reduced expression of *zif268* (an immediate early gene associated with long-term potentiation) in hippocampal subfields are associated with an impaired consolidation of aversive memory (41). In Alzheimer's disease, Braak's stages of neuropathological neurofibrillary tangles and plaques deposition are closely related not only to clinical symptoms but also to particular patterns of hippocampal neuron loss, mainly in CA1 and subiculum (42). While specific hippocampal subfields have been related to memory loss in several neuropsychiatric patients, their relevance in memory acquisition in non-neurological subjects remains an open quest. For instance, increased hippocampal volumes correlate with increased verbal IQ in adults (43), and DG and CA volumes correlate with verbal and visual memory (44).

Given the above-mentioned contribution of different hippocampal subfields to brain function, imaging studies using non-invasive MRI techniques with anatomically adjusted hippocampal annotation may unravel still unobserved clinical-pathological correlations. In fact, several studies have employed automatic, semiautomatic, and manual segmentation techniques to evaluate volumetric changes in the hippocampal subfields in conditions such as Alzheimer's disease, post-traumatic stress disorder, schizophrenia, major depression, epilepsy, and aging (45–51). However, most techniques fail to properly delineate hippocampal subfields, which can induce misleading conclusions. For example, several studies merge the hippocampal subfields in view of the difficulty of segmentation in clinical routine (44–49, 52–57). The difficulty in defining the hippocampal subfields in MRI also led other studies to only subdivide the subfields at the body level (47, 54, 58). Additionally, some segmentation protocols fail to follow the traditional subfield borders (14, 30, 59). For instance, the first version of the hippocampal segmentation algorithm of the FreeSurfer software (version 5.3), one of the most used automatic segmentation protocol (48, 52, 55, 56), estimated the CA2+CA3 volume as three times larger than the CA1 volume. This error occurs because of the significant portions of CA1, as defined by Duvernoy (30), being incorporated into the CA3+CA2 label, and as a result, CA2+CA3 volumes are overestimated while CA1 volume is underestimated. The latest version of the FreeSurfer software (version 6.0) updated the segmentation protocol closer to the histologically defined hippocampal subfields (29, 50). As a result, the CA1 volume in controls is 0.665 cm^3^ vs. 0.331 cm^3^ in the original version, while CA2+CA3 volume is now 0.213 cm^3^ vs. the previous 1.007 cm^3^. The new algorithm has a higher accuracy to discriminate Alzheimer patients from elderly controls (29) and improved the detection of HS in TLE patients (50). However, the FreeSurfer 6 hippocampal segmentation was unable to differentiate HS type 1 from HS type 2, in contrast to our manual segmentation. We searched retrospectively in our database for additional HS2 cases with presurgical MRI to increase the power of our finding. Even with four extra cases, HS type 2 remained undifferentiated from HS type 1. In addition, FreeSurfer indicates that the subfield with higher volume loss is DG+CA4 and not CA1, which is in disagreement with our manual segmentation and with the fact that both HS type 1 and HS type 2 present with higher neuron loss in CA1 (14). Although FreeSurfer detects hippocampal volume loss with good accuracy (60) and is a useful tool for whole brain analysis, the hippocampal subfield assessment should be interpreted with care in TLE patients, given its inability to discriminate HS types. In summary, our findings indicate that automatic hippocampal subfield assessments should not be used for distinguishing HS types in TLE. The variability in HS types, together with the fact that 3D T1-weighted images are often a part of the presurgical evaluation workup, make TLE an important model for testing any segmentation protocol, be it manual, semiautomatic, or automatic. Thus, we propose TLE patients that present different subtypes of HS as a calibration for the algorithms.

Our 4.7T data showed that DG+CA4, CA1, and the subiculum together account for 89% of the hippocampal volume. Compared with the other segmentation protocols, our results are similar to the studies of Malykhin's group (44, 49, 53). The use of manual delineation, Duvernoy's subfield definition (30), and grouping hippocampal subfields, indicated, in control subjects, volumes between 0.791 and 1.281 cm^3^ for DG, 1.115–1.713 cm^3^ for Ammon's horn, and 0.574–0.790 cm^3^ for the subiculum. Using the same grouping pattern, our delineation indicated a volume of 0.620 cm^3^ for DG + CA4, 1.660 cm^3^ for Ammon's horn, and 0.740 cm^3^ for the subiculum. However, a distinction of CA subfields is preferable for any meaningful correlation with clinical and neuropathological data. A very promising semiautomatic protocol developed with an *ex vivo* 9.4 T scanner also shows good similarities with our 4.7 T data in DG+CA4, CA3, and CA2 but not in CA1 (61). This is most likely due to problems with defining the CA1/subiculum borders, a fact clearly stated by the authors in the limitations section of the publication. Several studies have shown that higher field images (i.e., 4.7 T, 7 T, 9.4 T, and 10.5 T) improve the detection of subtle pathologies that were not detected in lower-field machines (1.5 T and 3 T with 1 mm^3^ voxels). For instance, 7 T images present with better anatomical definition than 1.5 T or 3 T images (62). Higher-field imaging can increase the detection of subtle pathological changes, such as mild malformations of cortical development, polymicrogyria, small focal cortical dysplasias, or other subtle changes (63, 64). Several studies showed that epilepsy patients with normal 3 T MRI can profit from a 7 T imaging, which improves the detection of pathologies in 21–70% of these cases (65–67). With regard to hippocampus, 7 T also improves its distinction from other close structures such as amygdala (68). Taking into account the recent FDA diagnostics approval and the anatomical quality, higher-field MRI are bound to become more widespread. So far, however, high-field scanners are restricted to few centers around the world.

Comparing TLE and controls, the hippocampal atrophy is correlated with neuron loss and volume loss in CA1 and DG. All cases from the present study had HS types with severe neuron loss in CA1 (i.e., HS type 1 and HS type 2). As for DG+CA4, this subfield is also severely affected in type 1 HS, but not in type 2 HS, which allowed us to differentiate these pathological entities. Thus, impact of DG+CA4 and CA1 neuronal density on the hippocampal volume was not unexpected in the present study. In a previous study with *in vivo* hippocampal volumetry, we showed that neuron loss and changes in extracellular matrix proteins in CA1 accounts to 38% of the hippocampal volume loss (9). A recent publication also indicated that high-field *ex vivo* MRI is able to differentiate HS type 1 from no HS based on Ammon's horn area and T2^*^ relaxation time (69). Moreover, rank correlation also showed significant associations between the HS class and Ammon's horn area, fractional anisotropy, T2^*^ relaxation time, and apparent diffusion coefficient (69). To our knowledge, no study so far has used different HS types as a way to evaluate the accuracy of their segmentation protocols. Our study indicates that, with a proper identification of subfield borders, HS type 1 can be distinguished from HS type 2. Further studies with a higher number of cases should confirm our findings and also evaluate if type 3 HS can also be distinguished based on volumetric assessment.

Some limitations of our study must be disclosed. First and foremost, we could not acquire whole hippocampus 4.7T MRI from our TLE cases, since we only received a section at body level from the surgical center. Thus, studies with MRI from whole hippocampi of TLE cases would be important to confirm our results. Although we evaluated TLE cases with 3T scanner, we considered our data as high-field because of the better resolution than regular clinical MRI. While clinical 3D T1-weighted images have isometric 1 mm^3^ voxels, our *ex vivo* image had voxels of 0.08 mm^3^, which allowed a better anatomical definition and a better distinction of subfield borders. As pointed by our previous study, other tissue components also account for the MRI changes in the hippocampus of TLE patients such as non-cellular elements of the extracellular matrix (9). However, owing to longer fixation of some samples, the immunohistochemistry for extracellular matrix and other tissue compounds were of poor quality for semiquantitative evaluation. Thus, the evaluation of these other components could improve the correlation between pathology and MRI volumetry. Another important point is the low number of type 2 HS. Since high-resolution data was collected prospectively, the cases that were evaluated followed the expected frequency of HS types. According to ILAE, the expected frequency of HS type 1 is 60–80%, 5–10% of TLE present with HS type 2, and 4–7.5% present with HS type 3. We collected twenty-two HS type 1 (88%), two HS type 2 (8%), and one HS type 3 (4%), which is not different from the expected proportions of HS types in TLE patients (exact test, p = 0.1). Since we only got one type 3, we excluded this case from the final manuscript. Thus, our findings must be confirmed by studies with a higher number of other HS subtypes. Even though we included extra HS type 2 cases to confirm the lack of discrimination of HS types by FreeSurfer's hippocampal subfield segmentation, a larger number of cases should be evaluated, including HS type 3, to confirm our observations. Finally, higher field MR (7T or higher) could further improve the definition of the borders between the hippocampal subfields in TLE.

In conclusion, our present study showed that DG+CA4, CA1, and the subiculum together account for almost 90% of all hippocampal volume. Comparing the evaluation of TLE cases confirms the importance of CA1 to hippocampal volume, and it highlights the importance of the CA4 subfield volume to distinguish HS type 1 from HS type 2, showing that neuron loss in these regions correlates with hippocampal volume loss. Finally, automatic subfield assessments in TLE should be interpreted with care and only trusted with matching histopathological data.

## Author contributions

JP-S, MM, and JL conceived and designed the experiments. JP-S, LdC, RS, and MM performed the experiments. JP-S, LK, PD, CG, AdS, and TV analyzed the data; JA, CC, CM, CG, AdS, TV, and MM contributed reagents, materials, and analysis tools. JP-S, PD, LK, LdC, and JL wrote the manuscript. JL, PD, LdC, RC, and IB critically reviewed the data, manuscript.

### Conflict of interest statement

The authors declare that the research was conducted in the absence of any commercial or financial relationships that could be construed as a potential conflict of interest.

## Supplementary material

Supplementary Figure 1 Staked tif file showing the anatomic boundaries of the hippocampal subfield throughout the hippocampal long axis in a 4.7T image (should be opened in ImageJ for full visualization). Blue, dentate gyrus + CA4; green, CA3; yellow, CA2; red, CA1; purple, subiculum.

Supplementary Figure 2 Hippocampal subfields, at the level of the body, in a control hippocampus imaged at 3T. Red, dentate gyrus + CA4; green, CA3; blue, CA2; cyan, CA1; magenta, subiculum.

Supplementary Figure 3 Automatic subfield evaluation with FreeSurfer of control cases (i.e., the contralateral hippocampi without evidence of HS; green boxplots) and TLE cases with type 1 HS (TLE-HS1, dark red boxplots) or type 2 HS (TLE-HS2, light red boxplots). TLE-HS1 patients had lower volumes, compared with controls, in all subfields, whereas TLE-HS2 had lower volume only in DG+CA4. The top portion shows z-scores, and the bottom graphs show absolute volumes (in mm^3^). The asterisks indicate difference from control cases, and the hash/pound sign indicates difference from TLE-HS1. DG, dentate gyrus; SUB, subiculum.

Supplementary Table 1 Intra-rater correlation coefficient. ^*^Pearson's correlation coefficient between evaluation 1 (JEP-S) and evaluation 2 (JEP-S with anatomical corrections from RC).
