# Supplementary figures and images for: Manual Hippocampal Subfield Segmentation Using High-Field MRI: Impact of Different Subfields in Hippocampal Volume Loss of Temporal Lobe Epilepsy Patients
Source: Front Neurol. 2018 Nov 20;9:927. doi: 10.3389/fneur.2018.00927 (PMC6256705; doi:10.3389/fneur.2018.00927)

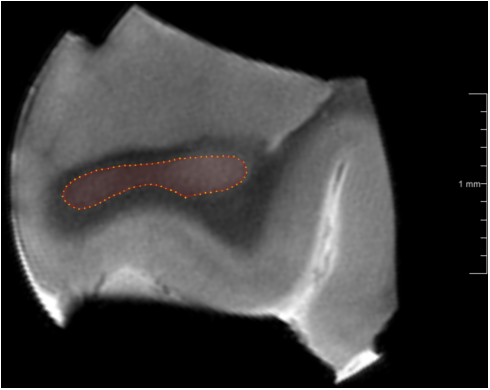

Supplement: Supplementary Figure 1 — Staked tif file showing the anatomic boundaries of the hippocampal subfield throughout the hippocampal long axis in a 4.7T image (should be opened in ImageJ for full visualization). Blue, dentate gyrus + CA4; green, CA3; yellow, CA2; red, CA1; purple, subiculum. [file Image_1.TIF]

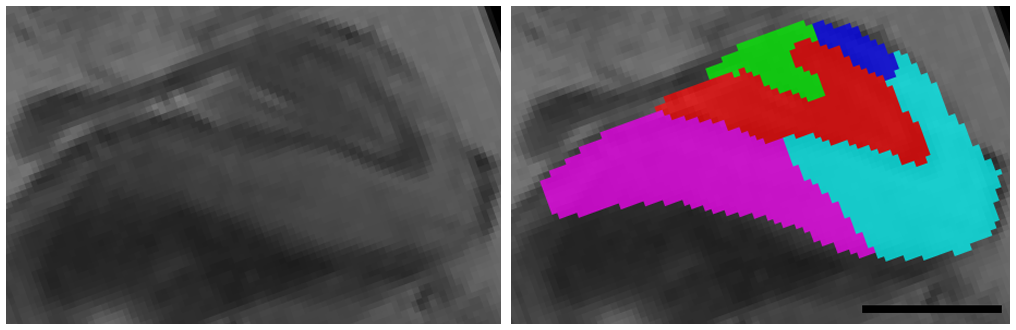

Supplement: Supplementary Figure 2 — Hippocampal subfields, at the level of the body, in a control hippocampus imaged at 3T. Red, dentate gyrus + CA4; green, CA3; blue, CA2; cyan, CA1; magenta, subiculum. [file Image_2.TIF]

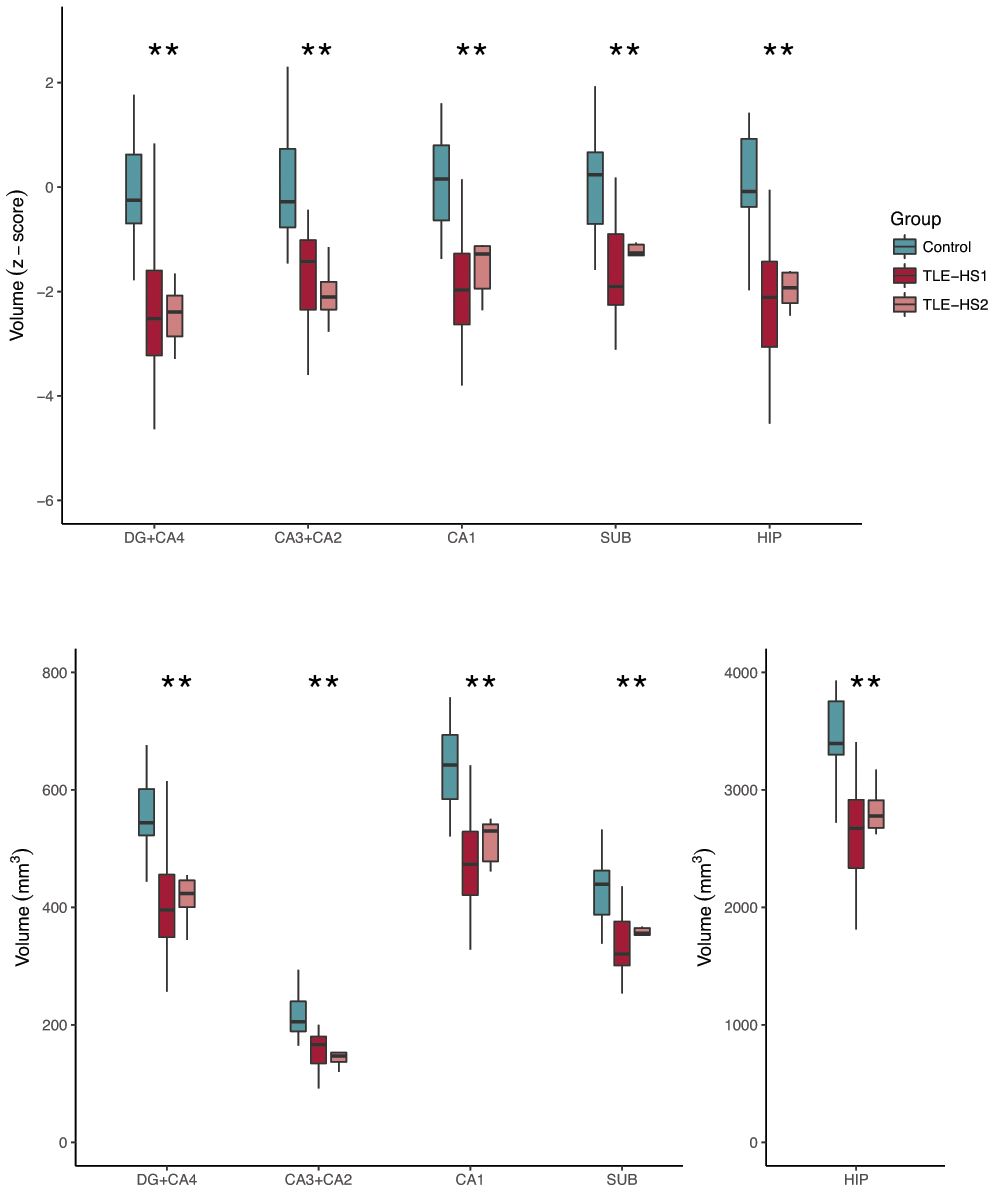

Supplement: Supplementary Figure 3 — Automatic subfield evaluation with FreeSurfer of control cases (i.e., the contralateral hippocampi without evidence of HS; green boxplots) and TLE cases with type 1 HS (TLE-HS1, dark red boxplots) or type 2 HS (TLE-HS2, light red boxplots). TLE-HS1 patients had lower volumes, compared with controls, in all subfields, whereas TLE-HS2 had lower volume only in DG+CA4. The top portion shows z-scores, and the bottom graphs show absolute volumes (in mm3). The asterisks indicate difference from control cases, and the hash/pound sign indicates difference from TLE-HS1. DG, dentate gyrus; SUB, subiculum. [file Image_3.TIF]
