# Supplementary material for: Manual Hippocampal Subfield Segmentation Using High-Field MRI: Impact of Different Subfields in Hippocampal Volume Loss of Temporal Lobe Epilepsy Patients
Source: Front Neurol. 2018 Nov 20;9:927. doi: 10.3389/fneur.2018.00927 (PMC6256705; doi:10.3389/fneur.2018.00927)
Supplement: Supplementary Table 1 — Intra-rater correlation coefficient. *Pearson's correlation coefficient between evaluation 1 (JEP-S) and evaluation 2 (JEP-S with anatomical corrections from RC). [file Table_1.docx]

**Supplementary Table 1.** Intra-rater Correlation Coefficient.

| Region | 3 T |  | 4.5 T |  |
| --- | --- | --- | --- | --- |
|  | R* | p | R | p |
| DG+CA4 | 0.997 | < 0.001 | 0.977 | < 0.001 |
| CA3 | 0.950 | < 0.001 | 0.948 | < 0.001 |
| CA2 | 0.976 | < 0.001 | 1.000 | < 0.001 |
| CA1 | 0.973 | < 0.001 | 0.960 | < 0.001 |
| SUB | 0.915 | < 0.001 | 0.878 | < 0.001 |
| HIP | 0.997 | < 0.001 | 0.960 | < 0.001 |

* Pearson’s correlation coefficient between evaluation 1 (JP-S) and evaluation 2 (JP-S with anatomical corrections from RC)
